# Hydrothermal Synthesis of MnWO_4_@GO Composite as Non-Precious Electrocatalyst for Urea Oxidation

**DOI:** 10.3390/nano12010085

**Published:** 2021-12-29

**Authors:** Patnamsetty Chidanandha Nagajyothi, Kisoo Yoo, Rajavaram Ramaraghavulu, Jaesool Shim

**Affiliations:** 1 School of Mechanical Engineering, Yeungnam University, Gyeongsan 38541, Korea; pcnnagajyothi@gmail.com (P.C.N.); kisooyoo@yu.ac.kr (K.Y.); 2 Department of Humanities and Sciences, Annamacharya Institute of Technology and Sciences, Rajampet, Kadapa 516126, India

**Keywords:** hydrothermal, MnWO_4_, graphene oxide, urea oxidation reaction (UOR)

## Abstract

In this study, manganese tungstate (MW) and MW/graphene oxide (GO) composites were prepared by a facile hydrothermal synthesis at pH values of 7 and 12. X-ray diffraction (XRD), scanning electron microscopy (SEM), transmission electron microscopy (TEM), X-ray photoelectron spectroscopy (XPS), and Raman spectroscopy were used for the structural, compositional, and morphological characterization of the nanoparticles (NPs). The XRD analysis revealed that the formation of monoclinic MnWO_4_ did not have impurities. The SEM and TEM analyses showed that the synthesized NPs were rod-shaped and well-distributed on the GO. The as-synthesized samples can be used as electrocatalysts for the urea oxidation reaction (UOR). The MW@GO-12 electrocatalyst exhibited higher current density values compared to other electrocatalysts. This study provides a new platform for synthesizing inexpensive nanocomposites as promising electrocatalysts for energy storage and conversion applications.

## 1. Introduction 

Urea (CO(NH_2_)_2_) is an abundant and easily available substance, which is found in human and animal urine and is widely used in the chemical industries and agriculture [1]. Urea-rich wastewater causes serious health issues like throat and lung irritation and environmental damages like eutrophication and acid rain [2,3]. However, urea is an efficient hydrogen (H_2_) carrier with non-flammable, non-toxic, and colorless properties [4,5,6]. Various traditional methods are used to treat urea-rich wastewater such as hydrolysis [7], adsorption [8], chemical oxidation [9], and biodegradation [10]; however, these methods are inefficient and expensive. Hence, it is important to develop an efficient, cost-effective, and convenient method to degrade urea-rich wastewater. The electro-oxidation of urea is essential in wastewater treatment and hydrogen production, solving both energy and environmental problems [11]. Noble metal-based catalysts are efficient catalysts for the urea oxidation reaction (UOR), but they are expensive, scarce, and limited to large-scale applications [12]. Hence, developing active, cost-efficient catalysts, which are easily available, is essential.

Manganese tungstate (MnWO_4_) is an inexpensive and eco-friendly material, and it has recently attracted attention owing to its high electronic conductivity, effective redox chemistry, and electrochemical, multiferroic, and ionic properties [13,14,15,16]. It is used in photocatalysis [17], electrocatalysis [18], gas sensors [19], and supercapacitors [20]. The use of transition metal oxides combined with carbon-based materials is a suitable way to improve the electrochemical properties [21,22,23]. Carbon-based materials play an important role in energy storage devices, transistors, sensors, etc., as they possess high physical, chemical, electrical, and thermal properties [24]. Among carbon materials, GO, the oxidized form of graphene, has a large surface area and displays strong hydrophilicity owing to the abundant oxygen-containing groups on its edges [25]. Furthermore, these functional groups (carboxylic, hydroxyl, and carbonyl) have been actively used to build novel composites. Mallick et al. synthesized cotton-fabric-derived mesoporous carbon-supported MnWO_4_ nanostructures for Zn-ion batteries [26]. Sardar et al. synthesized a MnWO_4_/amorphous CNT hybrid material for supercapacitor applications [27]. Xu et al. synthesized a GO/MnWO_4_ composite for magnetic resonance/photoacoustic dual-modal imaging and tumor photothermo-chemotherapy [28]. Jianhua et al. reported a layered MnWO_4_/rGO nanocomposite for supercapacitor applications [29].

In this study, we synthesized MW@GO at pH values of 7 and 12 by simple hydrothermal synthesis, and it had a nanorod-like morphology. It is extremely active in electrocatalytic urea oxidation under alkaline conditions. The XRD, TEM, Raman, and XPS results revealed that no impurity was observed between MnWO_4_ and GO. The hydrothermal synthesis is simple, rapid, and cost-effective, and the as-synthesized catalysts are inexpensive alternatives to precious metal catalysts for electrocatalytic UORs.

## 2. Materials and Methods

### 2.1. Synthesis of Electrocatalysts

All the chemicals used were of analytical grade and were obtained from Sigma-Aldrich. They were used directly without additional purification. Initially, GO (0.1 mg/mL) was sonicated for 1 h in a 50:50 *v*/*v* ratio of ethanol and DI water, and later Mn (NO_3_)_2_ (1.0 g) was added and stirred for 30 min to completely dissolve the salt. A second solution was prepared (50:50 *v*/*v* ratio of ethanol and DI water) and Na_2_WO_4_ (1.2 g) was also added and stirred for 30 min. The second solution (Na_2_WO_4_) was added dropwise to the first solution (GO-Mn (NO_3_)_2_) and stirred for 30 min. The ammonia solution was added until the pH reached 12, the reaction was shifted to a hydrothermal setup, and the temperature was maintained at 180 °C for 12 h. The mixture was allowed to cool naturally, after which the solid electrocatalyst was collected by centrifugation and dried at 70 °C. It was labeled as MW@GO-12 and left overnight. Other electrocatalysts were synthesized by the same method. The sample synthesized under neutral condition (pH 7) with GO was labeled MW@GO-7. The samples synthesized without GO at pH 7 and 12 were labeled MW-7 and MW-12, respectively. The characterization is presented in the Supplementary Information.

### 2.2. Electrode Preparation

An electrocatalyst slurry was prepared by mixing the electrocatalyst, acetylene carbon, and the binder (PVDF) at a ratio of 80:10:10 (wt. %) in 500 µL of NMP and sonicated for 30 min. The slurry was then drop-coated on the nickel foam and dried in a vacuum oven at 70 °C. Pt and Hg/HgO were used as counter and reference electrodes, respectively, and a whole electrochemical urea oxidation reaction was carried out in a 1.0 M KOH aqueous electrolyte with and without urea, at room temperature.

## 3. Results and Discussion

### 3.1. Characterization of Electrocatalysts

**Figure 1:**
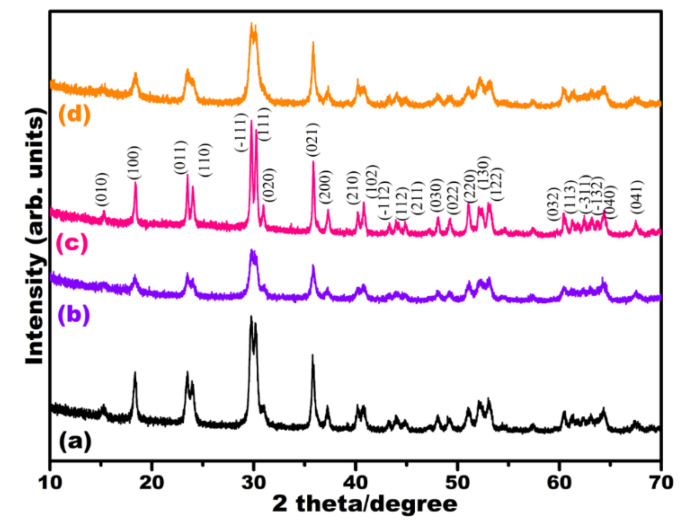
XRD patterns of the electrocatalysts, MW-7 (**a**), MW-12 (**b**), MW@GO-7 (**c**) and MW@.GO-12 (**d**).

Pure MnWO_4_ and MnWO_4_@GO were obtained at pH values of 7 and 12. At pH 7, the XRD peaks of the synthesized MnWO_4_ increased compared with MnWO_4_ at pH 12. Figure 1 shows the diffraction peaks of the MW-7 and MW-12 at 15.27° (010), 18.35° (100), 23.53° (011), 24.14° (110), 29.71° (−111), 30.18° (111), 30.99° (020), 35.83° (021), 37.23° (200), 40.12° (210) 40.79° (102), 43.28° (−112), 43.95° (112), 44.90° (211), 47.31° (030), 48.99° (022), 49.26° (220), 51.08° (130), 52.15° (122), 60.54° (032), 61.35° (113), 62.30° (−311), 63.83° (−132), 64.31° (040), and 67.47° (041). These values agree with the monoclinic crystal structure; space group: P2/c; space group number: 13 (JCPDS card no: 010-0477). Compared to pure MW-7 and MW-12 samples, there were no significant changes in MW@GO-7 and MW@GO-12 samples, and no noticeable diffraction peak of the GO was observed in these samples, as the regular stack of graphene oxide was destroyed by the intercalation of NPs [25].

**Figure 2:**
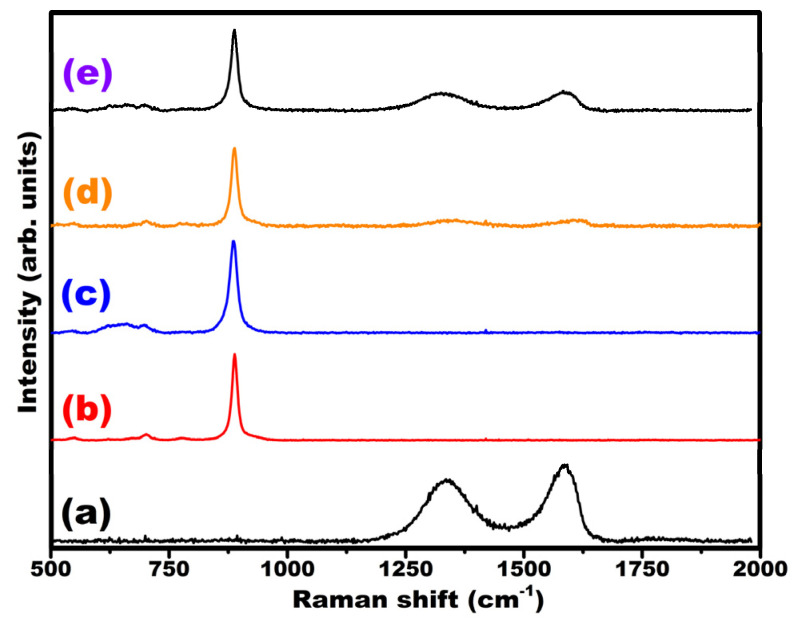
Raman analysis of the electrocatalysts, GO (**a**), MW-7 (**b**), MW-12 (**c**), MW@GO-7 (**d**), and MW@GO-12 (**e**).

Figure 2 shows the Raman spectra of the GO, MW-7, MW-12, MW@GO-7, and MW@GO-12. Pure GO exhibited the D and G bands at 1355 cm^−1^ and 1587 cm^−1^, respectively [30,31,32]. Pure MW-7 and MW-12 showed weak peaks at 548, 701, and 778 cm^−1^, which corresponds to the tensile vibration of Mn-O and the symmetric and asymmetric tensile vibration modes of W-O-W bonds, respectively [33,34,35]. The strong peak located at 889 cm^−1^ is related to the strong symmetrical stretching of the WO_2_ group in MnWO_4_ [35]. Both GO- and MnWO_4_-related bands were present in MW@GO-7 and MW@GO-12 samples. However, a slight shift in the D and G bands was observed in the composites. The Raman spectra results revealed the absence of the impurity phase in any of the synthesized catalysts. The UV-Vis spectra results are presented in the Supplementary Information (Figure S1).

**Figure 3:**
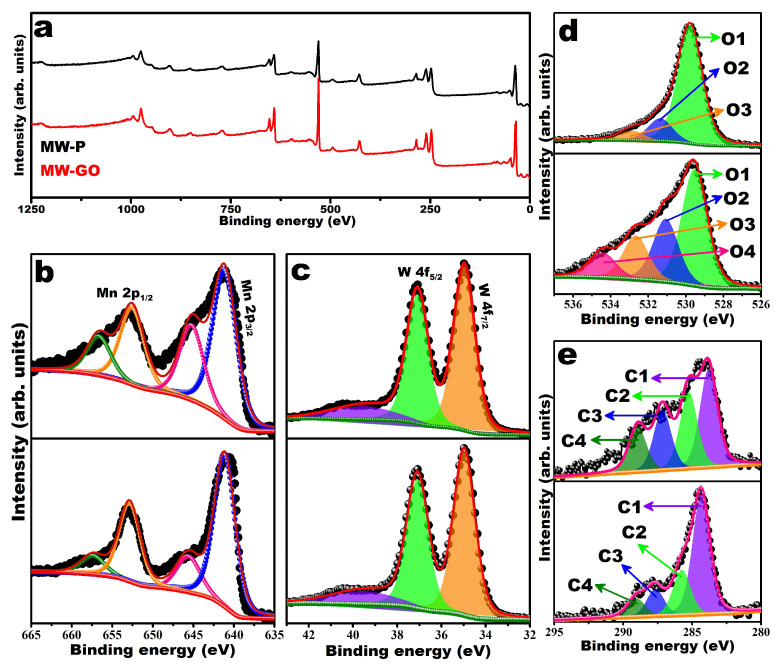
XPS analysis of MW-12 (above) and MW@GO-12 (below), survey scan spectrum (**a**), Mn-2p of MW-12 (above) and MW@GO-12 (below) (**b**), W 4f of MW-12 (above) and MW@GO-12 (below) (**c**), O 1s of MW-12 (above) and MW@GO-12 (below) (**d**), and C 1s of MW-12 (above) and MW@GO-12 (below) (**e**).

For an in-depth elemental analysis, the chemical nature was examined by the XPS as shown in Figure 3. Figure 3a shows the XPS survey spectrum of the hydrothermally synthesized MW-12 and MW@GO-12 composite, which showed the presence of Mn, W, O, and C. The synthesized MW-12 and MW@GO-12 were analyzed by a high-resolution XPS, and the Mn 2p spectrum was deconvoluted into four peaks (Figure 3b) with binding energies (BEs) of approximately 640.2, 645.3, 652.7, and 656.9 eV corresponding to Mn 2p_3/2_ and Mn 2p_1/2_, confirming the +2 oxidation state of Mn [26,36]. The W 4f spectra showed two peaks at 34.9 and 37.1 eV (Figure 3c), corresponding to W 4f_7/2_ and W 4f_5/2_, respectively, suggesting a +6 oxidation state of W [18]. The XPS spectra of as-synthesized MW-12 in the O 1s region showed three peaks at 529.4, 530.8, and 532. With 3 eV, however, the MW@GO-12 showed four peaks at 529.5, 531, 532.6, and 534.5 eV (Figure 3d). In both samples, the most dominant peak was observed at approximately 529 eV, associated with the Mn-O-W bond in MnWO_4_ and the remaining peaks related to O^−2^ present in the lattice of MnWO_4_, O=C-OH, and C-OH bonds, respectively [26,27,36,37]. The C1s spectra show four peaks at 284.2, 285.7, 287.6, and 288.9 eV (Figure 3e), corresponding to C=C/C-C, C-OH, C-O, and O=C-OH, respectively [26,38].

**Figure 4:**
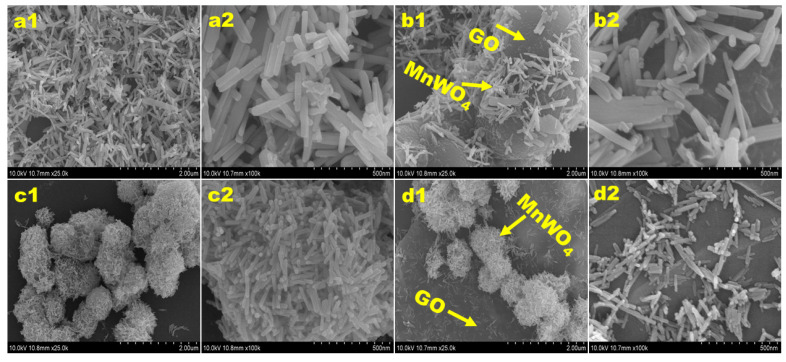
SEM image of hydrothermally synthesized electrocatalysts. MW-7 (**a1**,**a2**); MW@GO-7 (**b1**,**b2**); MW-12 (**c1**,**c2**); and MW@GO-12 (**d1**,**d2**).

**Figure 5:**
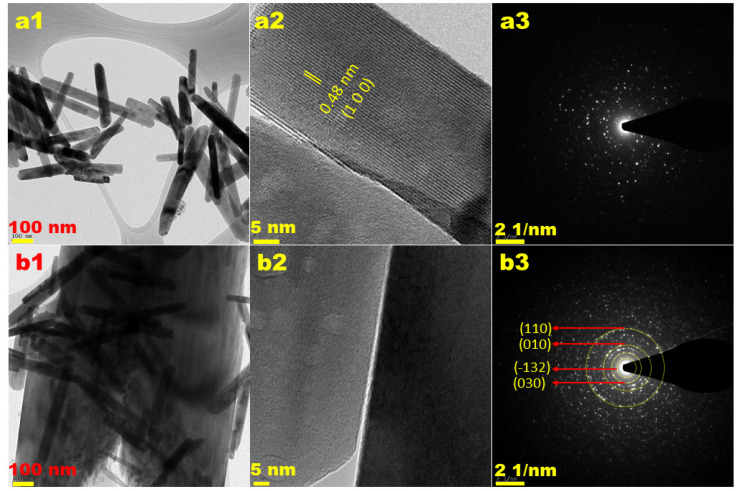
TEM analysis of electrocatalysts, low resolution MW-12 (**a1**), MW@GO-12 (**b1**), high resolution MW-12 (**a2**) MW@GO-12 (**b2**) and the corresponding SAED pattern MW-12 (**a3**), MW@GO-12 (**b3**).

Figure 4 shows the SEM images of MW-7, MW-12, MW@GO-7, and MW@GO-12 synthesized under different pH conditions. At pH 7, the synthesized MnWO_4_ was shaped as a rod (Figure 4(a1,a2)). As the pH increased from 7 to 12, the MnWO_4_ nanorods aggregated to a flower shape (Figure 4(c1,c2)). The SEM images of MW@GO-7 and MW@GO 12 are shown in Figure 4 b1 and b2 and Figure 4 d1 and d2, respectively. They did not show obvious changes in morphology when compared to pure MW-7 and MW-12 samples. The HR-TEM images of MW-12 and MW@GO-12 clearly show that the synthesized NPs had a nanorod shape and were randomly distributed on the graphene oxide nanosheets (Figure 5(a1,b2)). The d-spacing was measured using the Gatan software, and the results were shown in Figure 5(a2). The d-spacing of ~ 0.48 nm, which corresponds to the(100) plane. The SAED pattern reveals bright diffraction rings corresponding to the lattice planes of (110), (010), (−132), and (030), indicating that the synthesized NPs were crystalline in nature, which is well in line with the XRD results (Figure 5(a3,b3). The EDX analysis and elemental mapping results confirmed the presence of Mn, W, O, and C in the synthesized samples (Figure S2).

### 3.2. Urea Oxidation Reaction

**Figure 6:**
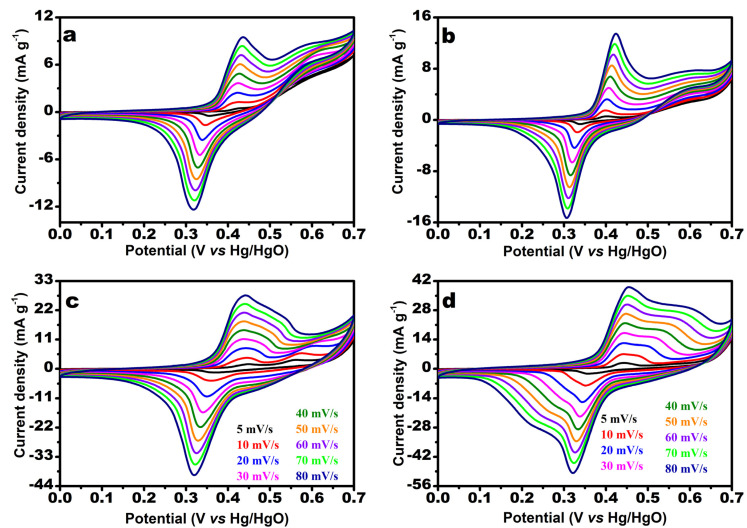
CV curves of the electrocatalysts at various scan rates; MW-7 (**a**), MW-12 (**b**), MW@GO-7 (**c**), and MW@GO-12 (**d**).

**Figure 7:**
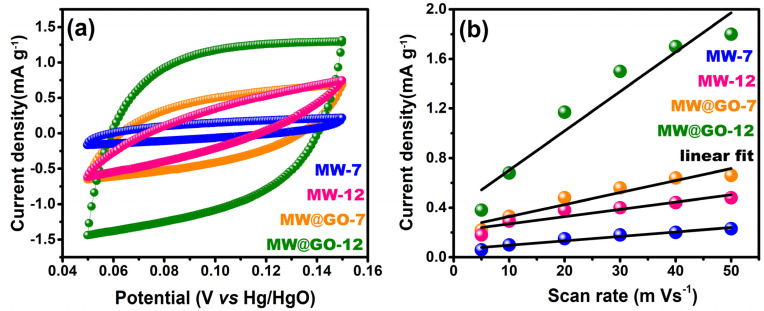
Comparative CV curves of the electrocatalysts for the ECSA analysis (**a**) and the corresponding electrochemical double-layer capacity linear fit plots of the electrocatalysts (**b**).

The electrochemical oxidation reaction of urea was investigated using a conventional three-electrode system with the CV and CA techniques. The CV curves of the synthesized electrocatalysts are shown in Figure 6; they were recorded at various scan rates (5–80 mV/s) in a fixed potential range (0.0–0.7 V) for Figure 6a–d, corresponding to MW-7, MW-12, MW@GO-7, and MW@GO-12, respectively. The CV curves showed clear redox peaks; there were no observable changes in peak shift with an increase in pH, although the peak sharpness increased (Figure 6a,b). Similarly, after the addition of GO, there were no observable changes in peak shift, although an enhancement in the current density was noticeable, which indicated that the addition of GO increased the current density of the electrocatalysts (Figure 6b,d). The surface coverage (Γ^*^) was calculated for the electrocatalysts [39], and the corresponding plot is shown in Figure S3a. The Γ^*^values of electrocatalysts MW-7, MW-12, MW@GO-7, and MW@GO-12 are 1.47 × 10^−7^, 1.94 × 10^−7^, 4.53 × 10^−7^, and 8.19 × 10^−7^, respectively. The Γ^*^ value increased rapidly with the addition of GO, and moderately with an increase in pH. The linear fit analysis was carried out for redox peak current densities vs. the square root of the scan rates for all electrocatalysts (Figure S3b). The linear fit plots suggest that the diffusion-control processes dominated the electrochemical studies. The electrochemical active surface area (ECSA) is a crucial parameter to identify the electrochemical performance. The ECSA was measured by the previously reported method [39], and the corresponding CV curves are shown in Figure S4. The double-layer capacitance (C_dl_) of electrocatalysts were 3, 5, 9, and 30 mF g^−1^ for MW-7, MW-12, MW@GO-7, and MW@GO-12, respectively (Figure 7a,b). From these values, MW@GO-12 electrocatalysts displayed superior ECSA. The addition of carbon will increase the catalytical active surface area. Sourav et al., reported that on introducing carbon into the electrocatalyst (MnWO_4_) the ECSA increased [26]; therefore, the ORR activity was also increased. Askari et al. developed ZnFe_2_O_4_-rGO nanohybrids, and, on adding GO, the ECSA was increased [40]. These reports suggest that the introduction of GO into the electrocatalysts will increase the ECSA.

**Figure 8:**
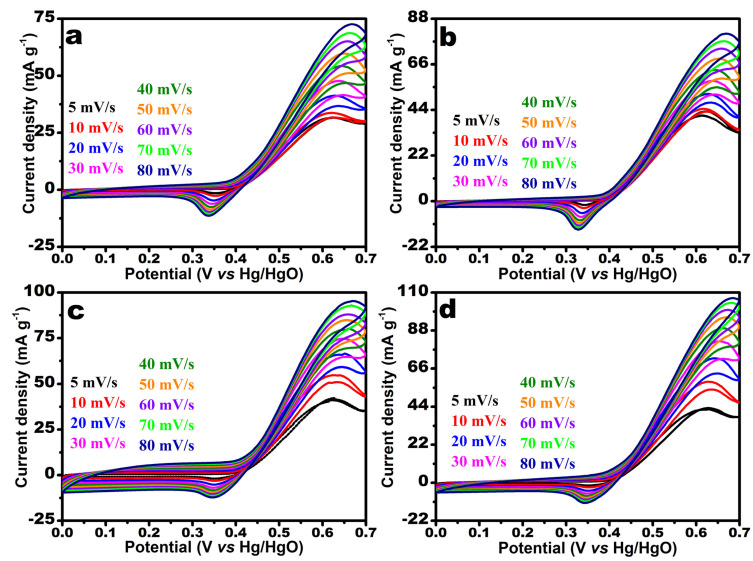
CV curves of the electrocatalysts of UOR analysis at various scan rates; MW-7 (**a**), MW-12 (**b**), MW@GO-7 (**c**), and MW@GO-12 (**d**).

The electro-oxidation of urea was performed using the electrocatalysts, and the results are shown in Figure 8a–d, labeled MW-7, MW-12, MW@GO-7, and MW@GO-12, respectively. UOR was carried out at various scan rates (5–80 mV/s) and a fixed potential range (0.0–0.7 V). The current densities of MW-12 slightly increased compared to MW-7 owing to the smaller size of the MnWO_4_ nanorods, whereas, on adding GO, the current density increased rapidly due to the higher electron transfer between GO and the electrocatalyst. The MW@GO-7 shows higher current densities compared to MW-7 and MW-12 due to the addition of GO. The MW@GO-12 electrocatalyst exhibited higher current density values compared to other electrocatalysts owing to its smaller size and the presence of GO [27,29]. Tang et al. synthesized layered MnWO_4_/RGOcomposite. They studied the supercapacitor application and noted a higher capacitance and stability compared to pristine MnWO_4_ [29]. To obtain more information on the kinetics reaction of UOR, the linear fit analysis was carried out for the anodic peak current densities of the electrocatalysts; its results are shown in Figure S5. The results suggest that the current densities increased linearly with an increase in the scan rate and that the UOR reaction process of all electrocatalysts was controlled by the diffusion-control process [41,42]. Electrochemical impedance spectroscopy (EIS) plays a vital role in understanding kinetic processes in electrochemical studies. Figure S6 shows the EIS plot along with the circuit and corresponding fit values of the electrocatalysts; it reveals that the MW@GO-12 electrocatalyst exhibited a moderate solution resistance, a lower charge-transfer resistance, and Warburg impedance compared to other electrocatalysts. These results also show that the MW@GO-12 electrocatalyst exhibited a higher catalytic performance.

The stability of the electrocatalysts in the UOR reaction plays a vital role, and, in this study, the CA technique was used to identify the stability of the electrocatalysts. Figure S7 shows the CA analysis of the electrocatalysts in the presence and absence of urea. The CA analysis was performed up to 9000 s after adding urea to the electrolyte, and the current density of the electrocatalysts increased compared to the pure electrolytes. The MW-7 electrocatalyst exhibited a lower current density but a higher stability compared to other electrocatalysts. The thermal stability of the synthesized MW-12 and MW/GO-12 catalysts was ascertained using TGA under the air atmosphere with a temperature ramp of 10 °C min^−1^ (Figure S8). The weight losses were observed above 700 °C, and the MW-12 and MW@GO-12 showed a 2.9% and 4.4% weight loss, respectively.

## 4. Conclusions

The facile hydrothermal-synthesized non-precious MnWO_4_/GO composites have been introduced as electrocatalysts for urea oxidation under alkaline conditions. The MW@GO-12 electrocatalyst exhibited higher current density values compared to the other electrocatalysts, i.e., MW-7, MW-12, and MW@GO-7. The enhanced electrocatalytic urea oxidation using MnWO_4_@GO composites revealed a high potential for future applications in wastewater remediation, fuel cells, and hydrogen production.

## Data Availability

Data can be available upon request from the authors.

## Supplementary Materials

The following are available online at https://www.mdpi.com/article/10.3390/nano12010085/s1. Figure S1: UV-Visible spectra of the electrocatalysts. Figure S2: EDS spectrum of MW@GO-12 electrocatalyst, and corresponding elemental mapping analysis. Figure S3: Current density vs. scan rate plots (a) and current density vs. the square root of scan rate plots of the electrocatalysts (b). Figure S4: CV curves of the electrocatalysts at different scan rates for electrochemical active surface area tests; MW-7 (a), MW-12(b), MW@GO-7 (c), and MW@GO-12 (d). Figure S5: Linear fit analysis of UOR for electrocatalysts, and the plots of peak current density vs. scan rate. Figure S6: EIS analysis of the electrocatalysts; inset shows the equivalent fitted circuit along with experimental data. Figure S7: Chronoamperometry analysis of the electrocatalysts in the presence and absence of the urea in 1.0 M KOH electrolyte. Figure S8. TGA analysis of pristine MW-12 and MW@GO-12 measured in air atmosphere.
