# Supplementary material for: Hydrothermal Synthesis of MnWO4@GO Composite as Non-Precious Electrocatalyst for Urea Oxidation
Source: Nanomaterials (Basel). 2021 Dec 29;12(1):85. doi: 10.3390/nano12010085 (PMC8746440; doi:10.3390/nano12010085)
Supplement: Supplementary file 1 [file nanomaterials-12-00085-s001.zip › nanomaterials-1494535-SI.pdf]

Article

Hydrothermal Synthesis of $MnWO_4$@GO Composite as Non-Precious Electrocatalyst for Urea Oxidation

Patnamsetty Chidanandha Nagajyothi [1], Kisoo Yoo [1], Rajavaram Ramaraghavulu [2,*] and Jaesool Shim [1,*]

[1] School of Mechanical Engineering, Yeungnam University, Gyeongsan 38541, Korea; pcnnagajyothi@gmail.com (P.C.N.); kisooyoo@yu.ac.kr (K.Y.)

[2] Department of Humanities and Sciences, Annamacharya Institute of Technology and Sciences, Rajampet, Kadapa 516126, India

* Correspondence: ramaraghavulu@gmail.com (R.R.); jshim@yu.ac.kr (J.S.)

Citation: Nagajyothi, P.C.; Yoo, K.; Ramaraghavulu, R.; Shim, J. Hydrothermal Synthesis of $MnWO_4$@GO Composite as Non-Precious Electrocatalyst for Urea Oxidation. *Nanomaterials* **2022**, *11*, x. https://doi.org/10.3390/nano12010085

Academic Editor(s): Genqiang Zhang and Shiqiang (Rob) Hui

Received: 19 November 2021
Accepted: 24 December 2021
Published: date

Publisher's Note: MDPI stays neutral with regard to jurisdictional claims in published maps and institutional affiliations.

Copyright: © 2021 by the authors. Submitted for possible open access publication under the terms and conditions of the Creative Commons Attribution (CC BY) license (https://creativecommons.org/licenses/by/4.0/).

Characterization

The morphology of the electrocatalysts was investigated using a field emission scanning electron microscope (SEM, S-4200, Hitachi, Japan), and a TEM (Tecnai G2 F20 S-Twin, USA) with an acceleration voltage of 200 kV in the Schottky mode. The crystal structures of the electrocatalysts were examined using XRD (PANalytical X'Pert³ PRO, USA) with Cu K_α radiation (λ = 1.54 Å). The elemental composition was quantitatively compared using XPS (K-alpha, Thermo Scientific, USA) with Al Kα radiation (1486.6 eV). Raman spectroscopy was performed using a Micro-Raman spectrophotometer (Horiba, USA). The absorbance spectrum of the electrocatalysts were obtained in the range of 200- 800, UV-Visible double beam spectrophotometer (VARIAN, Cary, 5000, USA).

Fig. S1 shows the UV-Vis spectra of MW-7, MW-12, MW@GO-7, and MW@GO-12. The MW-7 and MW-12 samples have broad and strong absorption peaks at about 400 nm and a weak bond at ~550 nm. In MW@GO-7 and MW@GO-12 samples, the weak band almost disappeared with the deposition of $MnWO_4$ NRs on GO.

SI-Figures

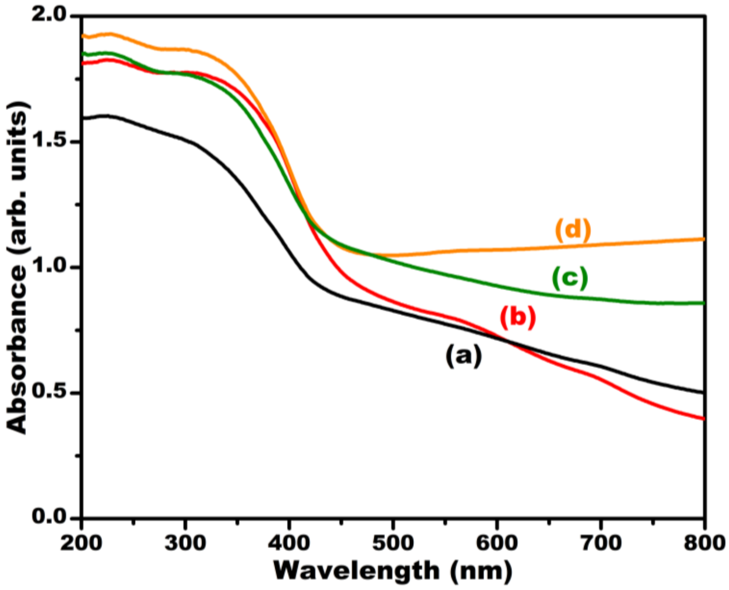

Figure S1. UV-Vis spectra of the electrocatalysts MW-7 (a), MW-12(b), MW@GO-7 (c), and MW@GO-12 (d).

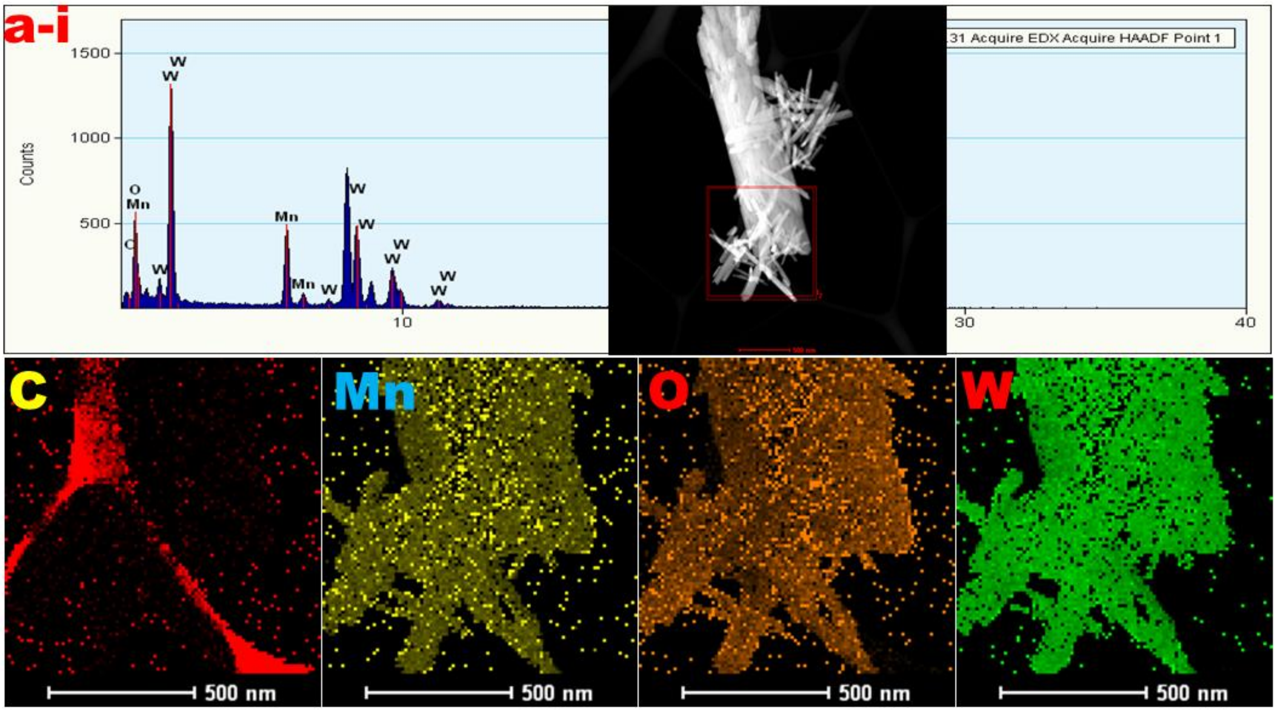

Figure S2. EDS spectrum of MW@GO-12 electrocatalyst, and corresponding elemental mapping analysis.

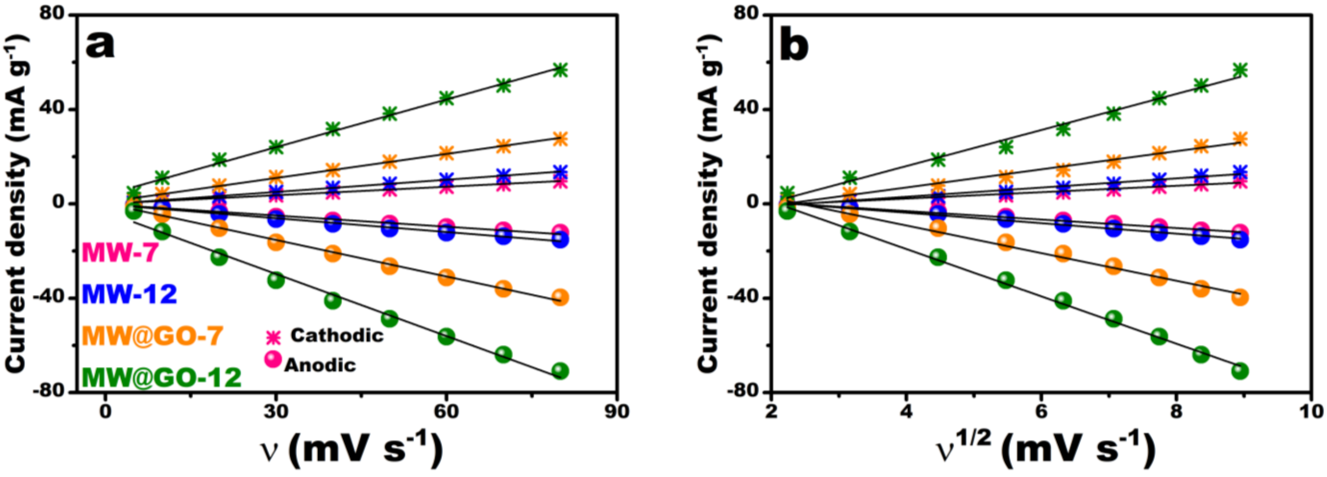

Figure S3. Current density vs. scan rate plots (a) and current density vs. the square root of scan rate plots of the electrocatalysts (b).

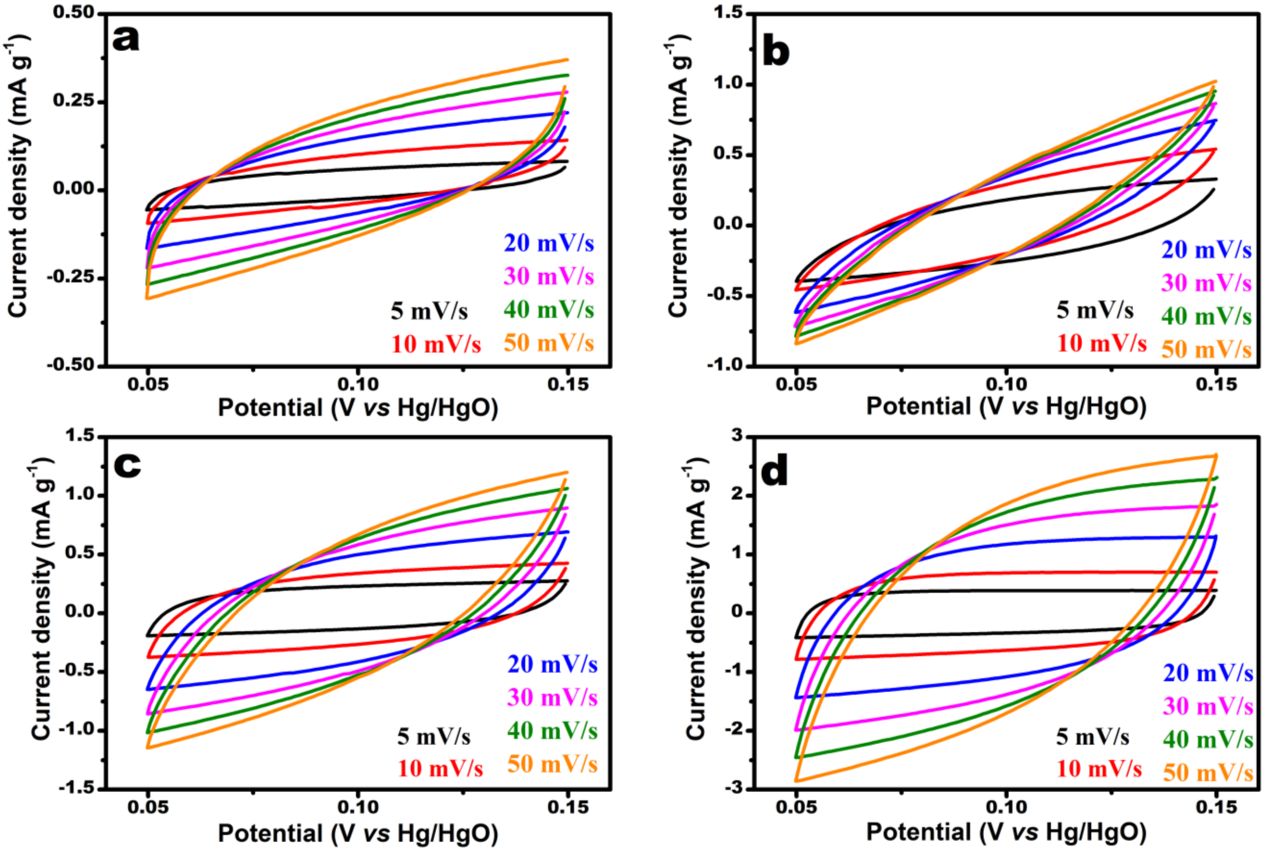

Figure S4. CV curves of the electrocatalysts at different scan rates for electrochemical active surface area tests; MW-7 (a), MW-12(b), MW@GO-7 (c), and MW@GO-12 (d).

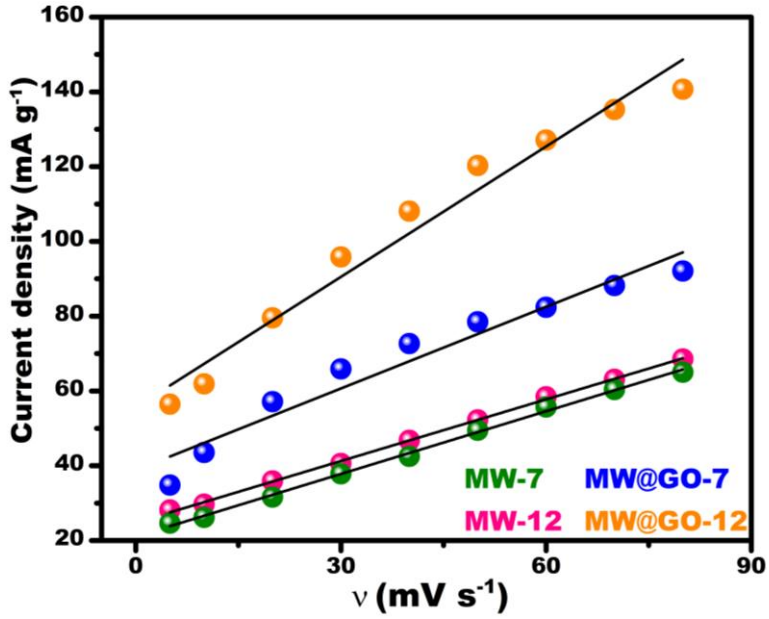

Figure S5. Linear fit analysis of UOR for electrocatalysts, and the plots of peak current density vs. scan rate.

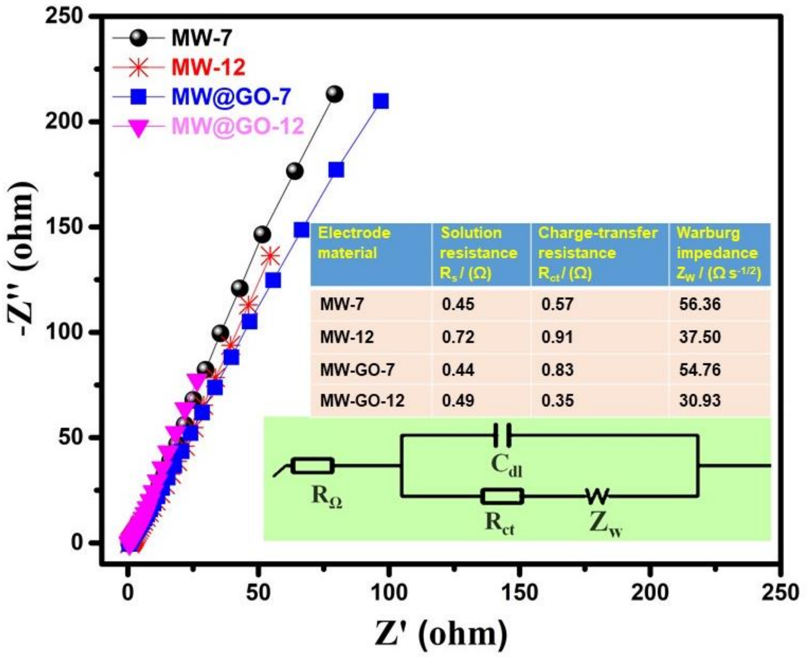

Figure S6. EIS analysis of the electrocatalysts; inset shows the equivalent fitted circuit along with experimental data.

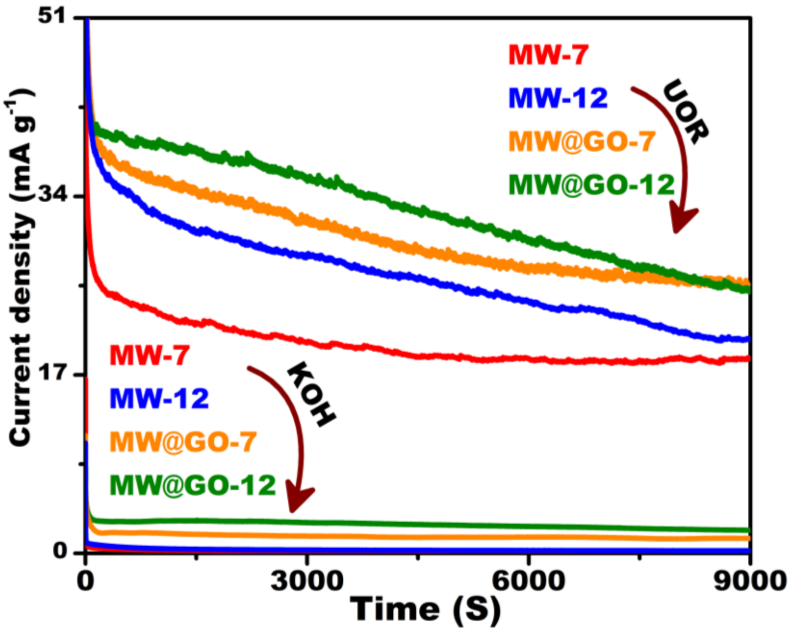

Figure S7. Chronoamperometry analysis of the electrocatalysts in the presence and absence of the urea in 1.0 M KOH electrolyte.

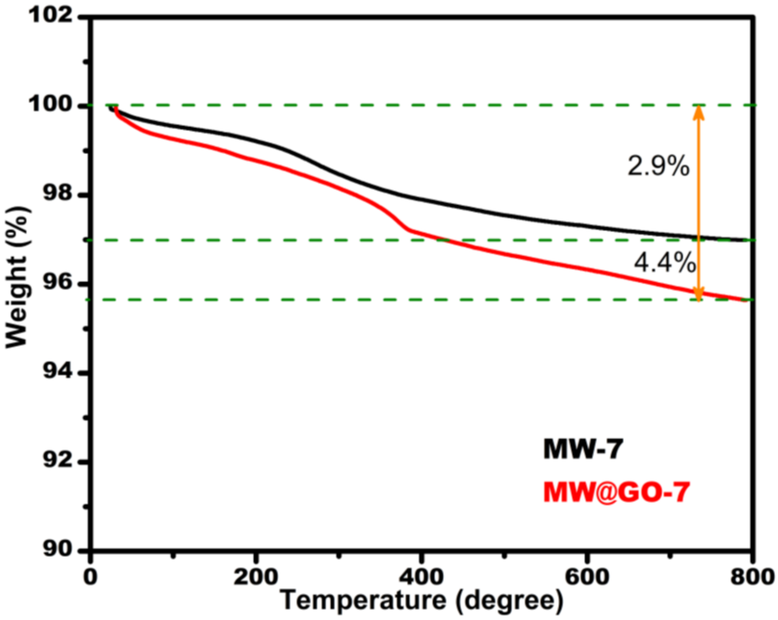

Figure S8. TGA analysis of pristine MW-12 and MW@GO-12 measured in air atmosphere.
